# 965. The Effects of Body Image and Itch on Sleep Quality in the Burn Population

**DOI:** 10.1093/jbcr/irag033.562

**Published:** 2026-04-06

**Authors:** Sean Wenzel, Huan Deng, Sheehan Choudhury, Serveh Mouloudi, Kara McMullen, Lynn M McCane, Suzanne Bertisch, Edward Santos, Shelley A Wiechman, Haig A Yenikomshian, Colleen M Ryan, Jeffrey C Schneider

**Affiliations:** Spaulding Rehabilitation Hospital, MA; Spaulding Rehabilitation Hospital, Harvard Medical School, Boston, MA; University of Massachusetts T.H. Chan School of Medicine, Worcester, MA; Spaulding Rehabilitation Hospital, Brighton, MA; Department of Rehabilitation Medicine, University of Washington, Seattle, WA; Spaulding Rehabilitation Hospital, Boston, MA; Brigham and Women's Hospital, Harvard Medical School, Boston, MA; Spaulding Rehabilitation Hospital, Charlestown, MA; University of Washington Harborview Medical Center, Seattle, WA; Division of Plastic and Reconstructive Surgery, Keck School of Medicine, University of Southern California, Los Angeles, CA; Massachusetts General Hospital, Harvard Medical School, Boston, MA; Spaulding Rehabilitation Hospital, Mass General Brigham, Harvard Medical School, Boston, MA

## Abstract

**Introduction:**

Recovery following burn injury is long and complex, with many biopsychosocial sequelae, including sleep. There is a gap in the literature exploring the association of itch and body image with sleep disturbance in the burn population. Therefore, this study aimed to understand the association between itch and body image and sleep disturbance among burn survivors.

**Methods:**

Data from a multicenter longitudinal database of burn survivors from 2015 to 2022 were analyzed. Demographic and clinical characteristics were compared between participants with no sleep disturbance (SD) (PROMIS SD Sleep Disturbance [SD] T-score < 55) and mild/moderate/severe SD (PROMIS SD T-score ≥ 55). Sleep disturbance was assessed primarily at 12 months. A logistic regression model quantified the association between itch and body image and sleep disturbance, adjusting for clinical and demographic variables, and uses robust standard errors to account for heteroskedasticity.

**Results:**

A total sample of 494 participants was included, with 368 self-reporting no SD, and 126 having mild, moderate, or severe SD at 12 months. Compared to the no SD group, the SD group was more likely to be younger (45.8 vs 48.1 median years, *p*=.048), non-Hispanic (84.1% vs 73.9%, *p*=.014), have longer acute hospitalization (23 vs 15 days, *p*<.001), unemployed (60.3 % vs 46.7%, *p*=.005), have public insurance (41.3% vs 26.1%, *p*=.005), have drug/alcohol abuse (15.9% vs 7.3%; *p*=.005), have pain medication use (44.4% vs 23.6%, *p*<.001), have received psychological therapy or counseling (31.8% vs 13.0%, *p*<.001), take psychiatric medications (47.6% vs 21.5%, *p*<.001), difficulty in hot environments (54.0% vs 29.1%, *p*<.001), and have a psychiatric history (11.9% vs 6.8%, *p*=.028).

Logistic regression analysis found higher odds of SD (OR = 1.18, CI = 1.06, 1.31; *p*=.003) for every one-point increase in an individual's itch score, and decreased odds of SD (OR = 0.55, CI = 0.39, 0.78; *p*=.001) for every one-point increase in an individual's body image score at 12 months, while holding the other variables constant.

**Conclusions:**

Sleep disturbance was associated with worse itch and body image at 12 months. The findings highlight the interconnectedness of these biopsychosocial outcomes after burn injury. Early identification and treatment of itch and body image issues may help clinicians manage sleep disturbance.

**Applicability of Research to Practice:**

Outpatient screening procedures for itch and body image at outpatient visits may help clinicians identify patients at risk for sleep disturbance and inform future multidisciplinary interventions.

**Funding for the study:**

Foundation Funding #90DPBU0008 and #90DPGE0004.

